# A Novel Perspective on the ApoM-S1P Axis, Highlighting the Metabolism of ApoM and Its Role in Liver Fibrosis and Neuroinflammation

**DOI:** 10.3390/ijms18081636

**Published:** 2017-07-27

**Authors:** Stefan Hajny, Christina Christoffersen

**Affiliations:** 1 Department of Clinical Biochemistry, University Hospital of Copenhagen, Rigshospitalet, Blegdamsvej 9, 2100 Copenhagen, Denmark; stefan.hajny.01@regionh.dk; 2 Department of Biomedical Sciences, Faculty of Health and Science, University of Copenhagen, Blegdamsvej 3, 2200 Copenhagen, Denmark; 3 Department of Cardiology, University Hospital of Copenhagen, Rigshospitalet, Blegdamsvej 9, 2100 Copenhagen, Denmark

**Keywords:** apolipoprotein M, Sphingoshine-1-Phosphate, lipid metabolism, liver fibrosis, blood brain barrier

## Abstract

Hepatocytes, renal proximal tubule cells as well as the highly specialized endothelium of the blood brain barrier (BBB) express and secrete apolipoprotein M (apoM). ApoM is a typical lipocalin containing a hydrophobic binding pocket predominantly carrying Sphingosine-1-Phosphate (S1P). The small signaling molecule S1P is associated with several physiological as well as pathological pathways whereas the role of apoM is less explored. Hepatic apoM acts as a chaperone to transport S1P through the circulation and kidney derived apoM seems to play a role in S1P recovery to prevent urinal loss. Finally, polarized endothelial cells constituting the lining of the BBB express apoM and secrete the protein to the brain as well as to the blood compartment. The review will provide novel insights on apoM and S1P, and its role in hepatic fibrosis, neuroinflammation and BBB integrity.

## 1. Introduction

Apolipoprotein M (apoM) was initially described by Xu and Dahlbäck in the late 1990s [1]. Hepatocytes are the major source of plasma apoM. Kidney and porcine Brain Capillary Endothelial Cells (pBCEC) also express and release apoM, their contribution to the overall plasma apoM pool is however elusive [2,3]. The corresponding apoM gene resides on chromosome 6 in the major histocompatibility complex class III region in humans and consists of a ~2 kb promoter sequence, 6 exons and 5 introns. To date, 5 SNPs in the apoM promoter region and 2 SNPs in the open reading frame (intron 5) are reported and associated with an altered lipid profile and various diseases such as diabetes, rheumatoid arthritis or cardio vascular disease [4,5,6,7].

The human apoM protein structure resembles a typical lipocalin consisting of an N-terminal α-helix, operating as signal peptide and anchor for lipoproteins, followed by eight anti-parallel β-sheets enclosing a hydrophobic binding pocket [8]. After post translational sialylation and/or *N*-glycosylation at Asn^135^ apoM emerges in one of its five isoforms differing in apparent weight (22.0 kDa–27.6 kDa) and isoelectric point (5.0–5.6) [8,9,10].

In contrast, structural analysis of mouse derived apoM revealed a highly unexpected, atypical lipocalin fold [11]. The binding pocket of mouse apoM consists of only seven anti-parallel β-sheets, which narrows the inner diameter of the lower part of the binding pocket by 3.7Å (−17% compared with human apoM) and thereby decreases the binding efficiency of long chain fatty acids.

Christoffersen et al. showed that apoM is the primary carrier for the small lipophilic signaling molecule Sphingosine-1-phosphate (S1P) [12]. The apoM/S1P axis will be discussed in detail and recent papers suggesting novel functionalities of the apoM/S1P complex will be addressed.

## 2. The Apolipoprotein M/Sphingoshine-1-Phosphate (ApoM/S1P) Axis—Introduction to the Concept

The majority (95%) of plasma apoM is bound to high density lipoproteins (HDL) and to a lesser extent to low density lipoprotein (LDL), very low density lipoprotein (VLDL) and chylomicron particles [13]. Due to its low plasma concentration (~0.9 µM) only ~5% of HDL and ~2% of LDL particles are estimated to carry apoM. Despite this, apoM significantly correlates with HDL as well as LDL and total cholesterol [14]. Generation and characterization of apoM knockout mice (apoM^-/-^) and transgenic mice with a 2-fold (apoM-TG^N^) and an 11-fold (apoM-TG^H^) increased plasma apoM level also constituted elevated plasma cholesterol levels by 13–50% in the apoM-TG strains and a 17–25% reduced cholesterol level in apoM^-/-^ mice. Two studies by Christoffersen et al. also revealed rapid apoM exchanged between HDL and VLDL/LDL particles [15,16]. Moreover, apoM enriched VLDL/LDL particles reduced clearance of VLDL/LDL from plasma in a LDL-receptor deficient mouse model. Thus, VLDL/LDL associated apoM may be involved in regulation of lipoprotein clearance from plasma.

Retinol and retinoic acid are classical ligands of lipocalins and initial experiments with apolipoprotein D (apoD) and apoM revealed that both apolipoproteins are able to bind these molecules (Table 1). Retinol can spontaneously dissociate from its binding partner, allowing the vitamin to freely move between compartments in vivo [17]. The lower binding affinity of retinol to apoM (K_D_: 2.2 µM) as to apoD (K_D_: 0.2 µM) or retinol binding protein (RBP; K_D_: 0.19 µM) suggests that the molecule may favor one of the latter [18,19,20]. Recombinant apoM binds sphingosine-1-phposhphate (S1P) with a K_D_ of ~0.9 µM, suggesting apoM as a primary carrier of the bioactive lipid [8,12]. Thus, apoM secreted by hepatocytes [21] or resident in blood plasma [12] correlates with plasma S1P levels [22]. Human serum albumin (SA) contains 3 binding sites for long-chain fatty acids [23] and binds S1P with a K_D_ of ~22 µM [24]. ApoM-deficient mice display 50% reduced plasma S1P levels, no detectable S1P in HDL, and unchanged S1P levels in the albumin fraction [12]. Despite the unchanged S1P-albumin concentration ApoM^-/-^ mice further displayed an increased endothelial permeability in the lung. This supports the hypothesis that apoM bound S1P is actively utilized, while albumin rather serves as a reservoir and scavenger for free S1P [12,25]. Interestingly, S1P shows a significantly lower binding affinity to HDL associated apoM (K_D_ 21 nM) as to LDL bound apoM (K_D_ 2.4 nM) [24] but also with a binding affinity significantly lower than previously reported for recombinant apoM [8]. Thus, a conformational change mediated by the lipoprotein phospholipid layer may enhance S1P recognition and binding to apoM. LDL particles are known to cause oxidation, inflammation and arterial lipid deposition promoting cardiovascular disease [26]. HDL on the other hand decreases LDL oxidation, improves endothelial function, stimulates cholesterol efflux from macrophages and is further associated with anti-inflammatory and anti-apoptotic effects [27].

Understanding of changes in the apoM/S1P axis and its biological relevance are limited. Silencing of apoM in mice significantly reduces cholesterol efflux from macrophages, increase cholesterol accumulation, and promotes development of atherosclerotic lesions [30,31]. Elsøe et al. further discovered that HDL associated apoM can protect against Cu^2+^ and AAPH (2,2′-azobis 2-methyl-propanimidamide, dihydrochloride) induced oxidation [32]. The lipocalin structure allows the protein to bind oxidized lipids and short-lived phospholipid oxidation radicals to hinder further oxidative processing. Whether these molecules displace or compete with S1P from apoM is however unknown.

The majority of S1P is intracellularly synthesized by sphingosine kinases (SPHK) [33,34,35] and passed on to its corresponding G-protein coupled receptors (S1P_1_–S1P_5_) by apoM or albumin [36]. Through autocrine and paracrine signaling pathways S1P exerts a significant role during development, ceramide synthesis, cell growth, survival and apoptosis, immune cell trafficking and lymphocyte differentiation [37].

Finally, S1P might also play a role in the progression of fibrosis [38,39]. While most studies identified S1P as a pro-fibrotic mediator, a recent study by Ding et al. demonstrated an improved hepatic regeneration post hepatectomy through treatment with apoM enriched HDL particles in mice [40]. It is, however, unclear whether S1P or apoM mediate the beneficial effects, as discussed later.

## 3. ApoM—Regulation, Modification and Release

ApoM gene expression is driven by various transcription factors such as Hepatocyte Nuclear Factor-1α (HNF-1α), Hepatocyte nuclear factor 3-β (FOXA2), apolipoprotein E (apoE) and Transforming Growth Factor β (TGF-β) [41]. TGF-β is an essential cytokine for cell growth, differentiation and apoptosis and also plays a pivotal role in immune cell differentiation [42] and fibrosis [43]. TGF-β also suppresses the apoM gene expression through TAK-1-JNK-c-Jun signaling which in turn reduces the apoM mRNA and protein levels by approximately 75% in a time and dose dependent manner in HepG2 cells [44].

Propofol, a routinely used anesthetic drug, increases the apoM expression levels by ~3-fold upon administration to HepG2 cells [45,46]. The concomitant increase of HNF-1α (2.5-fold) [45] or FOXA2 (7-fold) [46] may explain the observed effects. Hence, in a mouse model, *ip* injection of 10mg/kg propofol elevates the apoM protein content by a factor of 2.3 and HNF-1α by 2.5-fold. Taken together, these data support earlier studies where apoM levels in HNF-1α deficient mice where markedly reduced [30,47,48], and also sheds new light on the regulatory potential of propofol.

Lipoproteins and other apolipoproteins might also regulate the apoM gene expression. Kober et al. reported a 3.5-fold increase of apoM mRNA levels in pBCECs upon stimulation with HDL_3_ particles [3] and apolipoprotein E (apoE) knockout mice display a significantly increased apoM transcription rate together with elevated S1P plasma levels [49]. ApoE constitutes a crucial role in cerebral lipid metabolism and its potential suppressive effect on apoM may be an important mechanism in modulation of cerebral S1P distribution. A report by Christoffersen et al. further suggests that apoM containing HDL particles also carry apoE, which might further play a role in HDL catabolism through LDL receptor–related protein 1 (LRP1) and LDL receptors pathways [15].

The apoM release mechanism from hepatocytes, the proximal convoluted tubule of the kidney and pBCECs is unknown to date. Different studies identified the apoM signal peptide as one of the most determining structures probably controlling the process [50,51,52,53]. The hydrophobic tail, composed of 21 amino acids, anchors the apolipoprotein into the phospholipid layer of lipoproteins thus preventing renal clearance of apoM from plasma [54]. Studies in mice revealed that *iv* injection of recombinant human apoM^WT^ or apoM^22-188^, lacking the signal peptide, results in rapid clearance of apoM^22-188^ from plasma within ~2 h whereas ~50% of apoM^WT^ are still detectable [50]. Retention of the signal peptide is however an uncommon feature among apolipoproteins because it is typically cleaved off by a signal peptidase after translocation into the ER. ApoM lacks such a cleavage recognition sequence and only two other HDL associated proteins, Paraoxonase 1 (PON-1) and haptoglobin-related protein (HPR) also retain their signal peptide [55,56]. Introduction of an artificial cleavage site in the apoM signal peptide (apoM^Q22A^) revealed a higher secretion rate in vitro as when compared with native apoM [51,53]. Isolated extracellular fractions obtained from HEK293^apoM-WT^ or HEK293^apoM-Q22A^ cells contained only HDL associated apoM^WT^ or solitary apoM^Q22A^ [52], supporting the concept that apoM bioavailability relies on its attachment to lipoproteins. In HEK293 cells, the signal peptide refrains apoM in intracellular compartments and incubation with HDL accelerates apoM release [51]. A similar mechanism in cultured pBCECs has been reported by Kober et al., where HDL particles stimulated apoM export [3]. Brain derived HDL mainly constitutes of apoE [57], whereas apoA-I is predominantly associated with peripheral HDL particles. Hence, it would be intriguing to reveal whether polarized endothelial cells of the BBB respond to brain and peripheral derived HDL to the same degree.

Hepatocytes may release apoM during nascent pre-β-HDL formation. Pre-β-HDL particles represent a heterogenic HDL subpopulation consisting of pre-β_1_–pre-β_4_ particles differing in size, electrophoretic mobility and apoA-I content [58,59]. To date numerous studies highlighted the significant role of apoM in HDL biogenesis which has been recently summarized by Ren and Wroblewska [41,59]. In brief, apoM *per se* is not required to form pre-β-HDLs but larger sized particles originate only in presence of apoM [58]. Interestingly, a significant fraction of apoM is retained in intracellular compartments in HEK293 cells [51,58] and co-localizes with cholesterol in pBCECs [3]. Intracellular compartments such as the ER are however involved in cholesterol synthesis as well as in post transcriptional protein modification. Thus, a common synthesis pathway cannot be excluded and more detailed studies are demanded to pinpoint intracellular apoM depots.

## 4. ApoM Acts as S1P Scavenger in the Proximal Convoluted Tubule

ApoM is highly expressed in renal proximal tubule cells and secreted into the pre-urine by an unknown mechanism. Megalin, also known as LDL receptor related protein 2 (LRP2) is also expressed in proximal tubule cells and recognizes apoM among other lipocalins [54,60,61]. Interaction between apoM and megalin induces its internalization followed by degradation of apoM [50]. Thus, healthy mice excrete neither apoM nor S1P in the urine. In contrast, urine samples from LRP2^-/-^ mice comprise ~6 nM S1P and quantifiable amounts of apoM, suggesting megalin as a primary apoM receptor in proximal tubule cells [62]. The urinary loss of S1P does however not affect the respective plasma concentration, which suggests a unique role of kidney derived apoM.

Studies in an apoM^Q22A^ mouse model revealed low apoM plasma levels due to its inability to associate with HDL particles [50]. Free circulating apoM^Q22A^ will be rapidly filtrated by the kidney, followed by interaction with the megalin receptor, internalization and degradation. The hydrophobic signal peptide facilitates apoM solubility in apolar solvents and by the kidney excreted apoM emerges at a higher molecular mass [54]. This observation raises the question whether apoM associates with other intracellular (e.g., phospholipids or cholesterol) or extracellular factors to aid water solubility and probably export.

**Figure 1:**
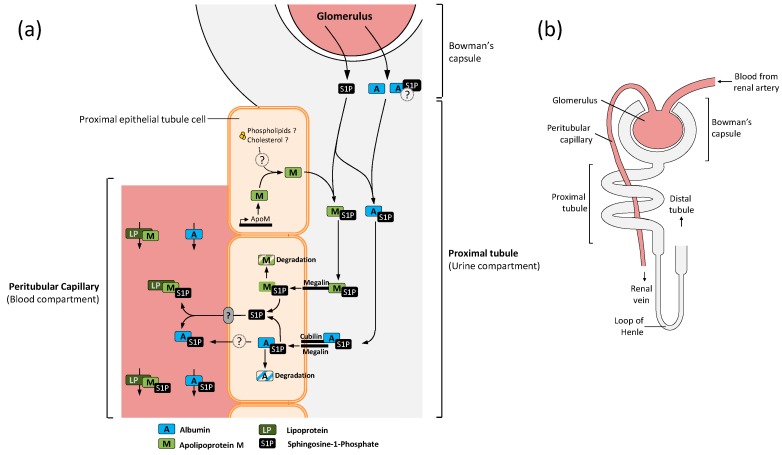
(**a**) Albumin and apoM operate as scavenger proteins in the proximal tubuli of the kidney to recover glomerular filtrated S1P. Whereas apoM is de novo synthesized and secreted by proximal epithelial tubule cells, albumin (probably already loaded with S1P) enters the tubuli through the glomerulus. Upon binding of free S1P, apoM interacts with the megalin receptor and albumin with its co-receptor cubilin, followed by internalization. A fraction of albumin may be further exported to adjacent peritubular capillaries (probably loaded with S1P molecules) whereas another portion undergoes protein degradation associated with release of scavenged S1P. Current evidence suggests that apoM will be rather degraded than exported. ApoM associated lipoproteins and/or albumin from peritubular capillaries further induce the export of accumulated S1P from the proximal tubule epithelium. These proteins are also involved in S1P binding and transportation to other cellular networks. (**b**) Macroscopic representation of the renal tubule system, highlighting the most relevant structures involved in apoM and albumin mediated S1P recovery.

We hypothesize that apoM and albumin are crucial S1P scavenger in the kidney, indispensable for S1P recovery (Figure 1). Indeed, albumin enters the proximal tubule via glomerular filtration [63] and may already carry S1P molecules. ApoM on the other hand is de novo synthesized and secreted by endothelial proximal tubule cells. To achieve solubility apoM is most likely intracellularly lipidated and/or associates with another soluble intra- or extracellular carrier protein. By an unknown pathway the complex is secreted into the pre-urine where apoM can bind free S1P followed by re-uptake via the megalin receptor. Whether apoM is recognized by other receptors downstream of the proximal tubule is however unknown to date and needs to be investigated in the future. If such an uptake occurs, we suggest that only minimal amounts of apoM are recovered. Megalin deficiency alters the plasma S1P levels only marginally [62] and thus a secondary transport system (probably located downstream of the proximal tubule) could be involved in apoM/S1P recovery from the pre-urine. Albumin reabsorption in the proximal tubule is mediated by cubilin, a co-receptor interacting with megalin [64]. It can however be only speculated whether albumin acts as an alternative S1P scavenger, notably due to its low S1P binding affinity [24] and putative occupation by other molecules. Renal uptake of apoM [50] and albumin [63,65] is probably accompanied by either lysosomal degradation and consequent release of scavenged S1P, or export to peritubular capillaries. Aseem et al. reported that cubilin haploinsufficiency results in decreased plasma albumin and apoA-I levels, thereby suggesting an export mechanism of scavenged proteins from proximal epithelial tubule cells to the adjacent blood vessels [66]. Surface plasmon resonance binding studies did however not show a response between apoM and cubilin, hence megalin is to date the only known receptor which interacts with apoM [54]. Whether apoM associates with apoA-I in the pre-urine, as documented for plasma apoM, to achieve solubility before or after release into the proximal tubule or post uptake via megalin is unknown to date. Thus, further studies are required to elucidate whether scavenged apoM is exported to the systemic blood flow by the proximal tubule endothelium. Lipoprotein associated-apoM as well as albumin from peritubular capillaries may further mediate S1P export, binding and transport to other cellular networks. Whether S1P *per se* acts as signaling molecule to steer the process is unexplored and needs to be addressed by further studies.

## 5. S1P Release from Primary Synthesizing Cells

The majority of circulating apoM likely origin from hepatocytes whereas a smaller fraction may derive from the blood brain barrier [2,3]. The vast majority of S1P is produced by vascular endothelial cells (EC), astrocytes and blood borne cells such as erythrocytes, platelets, macrophages and leucocytes, as summarized by Thuy et al [67]. It is, however, also evident that other cell types accumulate and release S1P, thereby also contributing to the systemic S1P pool [68,69]. Overexpression of apoM increases the intracellular S1P content in HepG2, HeLa and RAW264.7 cell lines, but only induces S1P release in HepG2 and HeLa cells. Consecutive mouse experiments with hepatic overexpression of apoM via adenoviral vector strategies revealed an elevated S1P content in liver and plasma. Whereas the expression levels of SPHKs (the key enzymes in S1P synthesis) were unchanged upon apoM overexpression, S1P degradation occurred at a lower rate, prompting the authors to suggest an apoM mediated inhibition of extracellular S1P degrading enzymes [69]. On the other hand, increased extracellular S1P levels implicate more apoM-S1P complexes and thereby protection against degradation.

Red blood cells (RBC) export S1P by an ATP dependent and vanadate and glyburide sensitive transporter [70]. RBCs constitute approximately 95% of total cells in whole blood and are therefore one of the main sources of plasma S1P [36]. A unique characteristic of RBCs is their capability to spontaneously release S1P without any known stimulus [70]. An extracellular S1P acceptor needs to be however present to facilitate effective S1P export. The assays of Kobayashi et al. revealed that the human serum albumin fraction is the most potent trigger to export ~1 nM S1P from human RBCs, followed by HDL (~0.5 nM), VLDL (~0.3 nM), LDL (~0.08 nM) and buffer (~0.06 nM). Specific incubation of RBCs with albumin or HDL showed that HDL triggers a ~85% higher S1P export than albumin [62,70]. Sub-analysis of HDL particles identified apoC-I and C-II as the most active components in the process [70]. Export assays with isolated apoC-I or C-II did however not induce S1P release. Moreover, phospholipid transfer protein (PLTP) might be additionally involved in S1P transfer from RBCs to HDL particles. Yu et al. detected by 60% decreased S1P plasma levels in PLTP knockout mice, thereby suggesting an essential role of the enzyme in the S1P transfer process [71]. More experiments are however required to elucidate the proposed pathway. Evidence of different in vitro experiments additionally point to the involvement of several ABC transporter in S1P release from RBCs [72,73]. Whereas a study by Kobayashi et al. suggests a vanadate (ABCA1 inhibitor) insensitive transporter [70], assays by Lee et al. however illustrated unchanged S1P plasma levels in ABCA1, ABCA7 and ABCC1 knockout mice [68]. Taken together, the exact mechanism that triggers S1P release from erythrocytes is still elusive to date.

Platelets are capable to store approximately 9 times more S1P than RBCs due to the absence of S1P degrading enzymes and maintenance of high SPHK activities [35,74]. Whereas platelets constitute only 5% of whole blood cells, RBCs constitute 45% suggesting that they are the primary source of S1P. It is furthermore considered that 54% of total blood S1P is located in erythrocytes, 32% in platelets and 14% in plasma (e.g., bound to albumin or apoM). Platelets release high amounts of S1P during blood coagulation and albumin has been identified as the preferred binding protein over apoM [75]. It is speculated whether this effect occurs due to apoM saturation or the requirement of an apoM-specific uptake mechanism. Aoki et al. also reported more pronounced S1P export from platelets in the presence of albumin than HDL (~60% less) [76]. To date, ABCA7 is stressed as the major S1P transporter in platelets due to its high abundance in this particular cell type [77]. ABCA7 knockout mice display however normal S1P levels [68]. Similar to RBCs, platelets also require an extracellular S1P acceptor to facilitate efficient S1P release. Platelets rely however on additional protein kinase C (PKC) activators to render S1P export by an ATP-dependent and glyburide-sensitive transport system or via a Ca^2+^ dependent pathway [70,78,79].

ECs are probably as crucial as RBCs in maintaining constant vascular S1P levels and may additionally render a compensatory function where S1P production from erythrocytes is insufficient [35,80]. ECs display structural and functional heterogeneity [81], thus S1P release mechanisms across the endothelium may differ. Numerous studies reported spontaneous S1P export from ECs in vitro and in vivo catalyzed by transporters of the ABC family and/or Sphingolipid Transporter 2 (SPNS2) [36]. A recent study illustrates that apoA-I is sufficient to induce S1P export from cultured HUVECs and provides novel evidence that ABCA1 might play a more distinctive role in S1P export form ECs as previously assumed [82]. It is, however, unclear whether the export experiments were conducted using FCS free medium (albumin and HDL free). We assume that an acceptor can be crucial to capture/bind released S1P thereby acting as a chaperone to prevent its degradation and to facilitate transportation. A unique feature of ECs comprises the expression of SPNS2 to render supplementary S1P export to blood and lymph [35,83,84,85]. SPNS2 deficient mice display by 23% reduced plasma and by 86% decreased lymph S1P levels [86]. Thus, SPNS2 might play a major role in S1P release to lymph, whereas export into the plasma compartment appears to be auxiliary. Additional studies are however required to identify the exact transport mechanism and to elucidate whether apolipoproteins and/or albumin interact with SPNS2 to induce S1P secretion.

## 6. S1P Release Mechanism from ApoM

Albumin and apoM are the only known proteins to bind and transport S1P in the circulation. HDL associated apoM carriers around 65% of plasma S1P [12] and is considered to be a provider of actively used S1P whereas albumin-S1P serves as a reservoir.

S1P can bind five different receptors (S1P_1_–S1P_5_) and only the structure of S1P_1_ has been resolved by protein crystallization [87,88]. As previously discussed, apoM-bound S1P and albumin-bound S1P might serve different roles. Whereas apoM bound S1P induces S1P_1_ internalization and recycling to the plasma membrane, albumin triggers S1P_1_ internalization followed by proteasomal degradation upon binding [89,90].

In silico, studies suggest that the upper section of the apoM binding pocket (calyx) can switch between an open, probably ligand accepting, and a more closed conformational state [91]. Via steered molecular dynamics simulations spontaneous S1P release is unlikely since the estimated energy of unbinding is higher than 60 kJ/mol. A tight interaction between apoM and a S1P receptor (or a cofactor) is probably necessary to decrease the energetic barrier to pass on S1P through the calyx.

**Figure 2:**
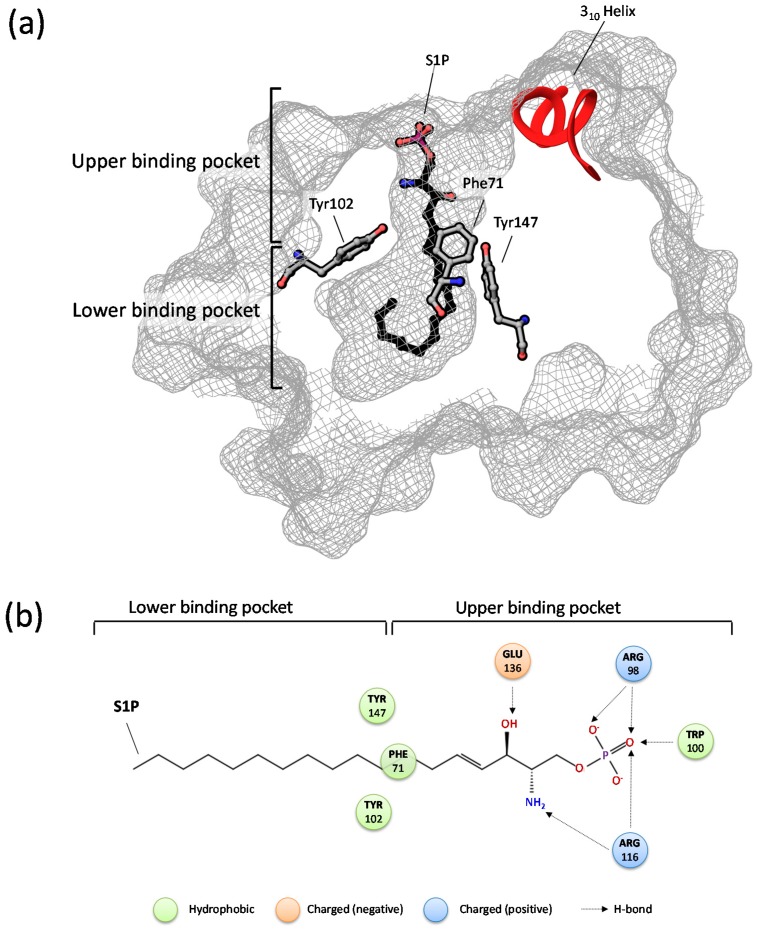
(**a**) Schematic representation of human apoM in complex with S1P. The amino acid residues Phe71, Tyr 102 as well as 147 play a leading role in separating the hydrophobic binding pocket into a lower and upper section. The 3_10_ helix may furthermore play a significant role in recognition and binding of small molecules. (**b**) The upper part of the binding pocket exerts a critical role in fixating the S1P phosphate head in apoM via Arg98, Trp100, Arg116 and Glu136 interaction.

Amino acid residues Arg98, Trp100, Arg116, and Glu136 in human apoM stabilize the charged S1P phosphate head and thereby constitute the major energetic limitation to release the molecule (Figure 2) [12]. Moreover, Tyr102 and Tyr147 are highly flexible in ligand free apoM and are thereby potentially involved in shielding the lower part of the binding cavity together with Phe71 in the absence of S1P [91]. In parallel, a significant wider diameter of the upper binding cavity which (most probably) supports ligand recognition and binding was observed. Comprehensive analysis of the crystal structure further highlights the apoM 3_10_ helix as crucial element for ligand recognition and probably apoM-protein interactions.

A second study revealed that both, HDL associated and recombinant apoM, are able to deliver S1P to S1P_1_, as visualized by stimulation of chemo attraction in HUVECs [12]. Crystallization of apoM further elucidated high flexibilities within the first β-strand. Hence, changing its conformational state may open the lower part of the binding pocket, thereby promoting S1P release.

Structural analysis of S1P_1_ revealed that a direct transmission of S1P from apoM to the receptor is unlikely [87,88]. S1P has to be deposited into the outer leaflet of the cellular membrane for a lateral move into the receptor binding pocket, induced by a conformational change of S1P_1_. Thus, a direct interaction between apoM (or another HDL associated protein) and S1P_1_ may mediate S1P transmission from apoM into the cellular membrane.

Based on a study in HUVECs by Liu and colleagues, HDL binds to its receptor Scavenger receptor class B member 1 (SR-BI) via apo-AI [82]. The binding contributes to S1P_1_ activation through HDL-S1P, which in turn induces intracellular pathways such as S1P synthesis. It is well known that SR-BI mediates bidirectional unesterified cholesterol movement indispensable for HDL maturation and remodeling [92]. It is, however, unknown whether apoM per se interacts with particular cellular (co-)receptors upon HDL-SR-BI binding in order to release S1P. HDL containing apoM induces however more efficient cholesterol efflux than HDL without apoM, thereby pointing to higher binding affinities in the presence of apoM [93].

## 7. The Role of ApoM in Liver Fibrosis

Over the last decade S1P has emerged to one of the most pivotal signaling molecules in hepatic tissue regeneration and misguided wound healing ultimately leading to liver fibrosis [94,95]. Per definition, fibrosis is the accumulation of fibrous connective tissue in damaged or inflamed organs resulting in permanent scarring, malfunction and/or death [96,97]. Liver fibrosis occurs as a precursor of cirrhosis due to chronic liver disease, severe injury or dysregulated wound healing. In 2012 approximately 35% of the male and 16% of the female population suffered of liver cirrhosis worldwide [98].

Hepatocytes can restore non-severe liver injuries under physiological conditions [99]. More serious damages however exceed the repair capabilities resulting in substitution by extracellular matrix (ECM) proteins and inflammation. Hepatic stellate cells (HSC) reside in the space of disse and play a critical role in the progress of liver fibrosis, as reviewed by Zhang et al. [100]. In brief, severe liver damage leads to HSC activation which in turn transdifferentiate to proliferative and contractile myofibroblasts. Activated HSCs further express glial fibrillary acidic protein (GFAP), matrix metalloproteinases (MMPs) and tissue inhibitors of metalloproteinase (TIMPs). Secretion of collagen type I and III and elevated synthesis of alpha smooth muscle actin (αSMA) are also reported [101]. Accumulation of reactive oxygen species (ROS) and apoptotic cells can further promote HSC mediated ECM aggregation and chronic inflammation, ultimately leading to scar formation [99,100]. In addition to HSCs, bone marrow derived cells are able to migrate to fibrotic liver tissue, transdifferentiate into myofibroblasts and also contribute to progression or regression of liver fibrosis [102].

A common experimental model of liver fibrosis comprises surgical removal of vital liver tissue by up to 90% of (partial hepatectomy) [103]. Common bile duct ligation (BDL) on the other hand causes periportal biliary fibrosis, cholestasis, and hyperproliferation of biliary epithelial cells promoting expression of fibrogenic markers such as TGFβ1, α-SMA, TIMP-1 and α-SMA, consequently involved in ROS generation and hepatic damage, whereas carbon tetrachloride (CCl_4_) injection chemically induces liver fibrosis via promotion of lipid peroxidation, free radical reactions, necrosis of centrilobular hepatocytes, inflammation, and liver fibrosis [104,105].

A recent study suggests that apoM is involved in liver regeneration via modulation of LSEC proliferation [40]. LSECs excerpt anti-fibrotic actions and possess a vital role in liver regeneration post traumatic injury [40,106,107]. Also, S1P plays a significant role on sinusoidal protection against experimentally induced apoptosis [108], and stimulates proliferation of hepatocytes via IL-6 and VEGF signaling [109]. Secondly, a direct interaction between TMNK-1 cells (immortalized LSEC) and platelets is needed to promote hepatocyte proliferation through S1P mediated IL-6 release [110]. A consecutive approach by Matsuo reproduced these finding in a 70% hepatectomized rat model [111]. They infused rhodamine-6G labelled platelets from syngenic rats (10% of total circulating platelets) into hepatectomized animals and observed rapid accumulation of the labeled cells in liver sinusoids. Hence, S1P induced liver regeneration can also be mediated in a carrier independent manner. Thus, a direct physical interaction between LSECs and platelets may be additionally considered.

ApoM knock out mice exhibit a severe vascular maladaptive remodeling phenotype in their hepatic sinusoidal vasculature after either 70% hepatectomy or BDL [40]. The animals displayed markedly increased SMA protein and collagen expression levels in their liver after BDL, whereas apoM-TG (by 11-fold increased apoM expression) and control mice exhibited distinctive lower levels. Additional experiments in an endothelial cell specific S1P_1_ knock out mouse model recapitulated these findings, pointing to S1P_1_ as the critical S1P receptor in mediating LSEC recovery and further liver regeneration.

Controversially, S1P_1_ signaling has been associated with inhibition of sprouting angiogenesis in various reports [112,113] and other studies illustrated fibrotic progression upon S1P mediated S1P_1_-S1P_3_ signaling in BDL or CCl_4_ models [39,114,115,116]. Surgical excision of vital liver tissue eliminates different cell types such as hepatocytes, sinusoidal endothelial cells, Kupffer cells and HSCs which in turn induces an additional inflammatory reaction. The observed effects may be confounded by the chosen model system and further studies are necessary to comprehensively characterize each of them.

Bile acids are important components of liver injury and liver fibrosis. It is unknown whether de novo synthesized or from the periphery imported apoM (probably carrying S1P) is exported into the bile juice and whether the complex promotes pro- or anti regenerative processes. It has been however documented that various apolipoproteins such as apoA-I and A-II exert a similar role in the bile as in the periphery [117,118]. Both, S1P and apoM induce liver receptor homolog-1 (LRH-1) expression suggesting a possible pathway of regulating bile acid metabolism [119,120]. Whether apoM is actively secreted into the bile juice (in complex with or without S1P) needs to be investigated in the future. Moreover, a recent publication highlighted the role of S1P_2_ in cholestasis-induced liver injury in mice [121], thereby suggesting a role of the apoM/S1P complex in that process.

Nevertheless, the controversial findings between HDL-apoM and S1P receptor mediated effects on liver regeneration suggest that HDL associated apolipoprotein(s) may play a unique role in tissue regeneration post traumatic injury. ApoM may exert such a role, even though more experiments are needed to clarify the mechanisms.

## 8. ApoM May Exert an Essential Role in Cerebral S1P Transport

The blood brain barrier (BBB) is a multicellular network isolating the central nervous system from the peripheral circulation [122]. The cooperation between astrocytes and endothelial cells possess a pivotal role in regulating the passage of ions and molecules through the BBB to facilitate physiological neurotransmission and to protect the central nervous system (CNS) from pathogenic substances. In contrast to the peripheral endothelium, ECs of the BBB lack fenestration and develop continuous intercellular tight junctions [122,123]. Such properties reduce the transcytosis rate and thereby facilitate highly selective import of oxygen, nutrients and other molecules, concurrent with export of toxins, pathogens and various brain derived products.

A major hallmark of CNS related diseases comprises a disrupted BBB. Local accumulation of inflammatory mediators is accompanied with elevated expression of selectins, adhesion molecules and chemokines further promoting leukocyte migration through the endothelium into the brain parenchyma [124].

The role of S1P in the process of inflammatory pathologies in the CNS has been investigated by numerous groups over the last decades [125,126] and beneficial effects of the S1P analogue FTY720 in animals with experimental multiple sclerosis have been reported [127,128,129,130]. For instance, FTY720 reduces recirculation of autoreactive lymphocytes to the CNS and alters trafficking and function of B-cells as well as natural killer cells [130,131].

In vivo experiments in model systems resembling cerebral inflammatory processes exploring the role of S1P and its chaperone apoM are essentially elusive. Blaho et al. investigated the effect of apoM in a mouse autoimmune encephalomyelitis (EAE) model resembling multiple sclerosis [132,133]. ApoM deficient EAE mice develop more serious CNS pathologies as WT animals and apoM^TG^ rodents with a by 11-fold elevated apoM expression displayed an even milder manifestation. Thus, the apoM-S1P axis might play an essential role in cerebral immunetrafficking and thereby exerts a protective function against autoimmune inflammatory pathologies.

An interesting study by Kober and colleagues revealed native apoM gene and protein expression in cultured pBCECs [3]. Moreover, the whole porcine brain comprised a by ~80% higher apoM mRNA content than pBSECs. Hence, ECs at the BBB are most probably not the only source of CNS localized apoM. Astrocytes are known to synthetize apolipoproteins such as apoE [134], apoD [135] and the LDL associated glycoprotein apoJ [136,137], also known as Clusterin [138]. It is, however, unknown whether these cells also express apoM. In contrast to plasma where apoA-I and apoB are the most pronounced apolipoproteins in lipoproteins, astrocyte derived apoE is the most abundant apolipoprotein in the CNS where it regulates cerebral lipid metabolism and formation of HDL-like particles together with apo-AI and apoJ [57,139]. In the brain residing apoA-I is however not synthesized by CNS associated cells but enters the organ via the blood–cerebrospinal fluid barrier and probably to a lesser extent via the BBB [140]. The transport mechanism is unknown. It is, however, intriguing that ABCA1^−/−^ mice display a 50% reduced cerebral and a 85% reduced peripheral apoA-I level [141]. In parallel, CNS specific ABCA1 knockout increases the brain apoA-I content by a factor of 4 [142]. The majority of SR-BI localizes at the apical (to the blood faced) membrane and selective HDL uptake at the BBB via SR-BI has been reported [143]. It can be however not excluded that apoA-I and HDL (with apoM) can cross the BBB. Whether SR-BI and/or ABCA1 are also involved in basolateral export needs to be elucidated in the future.

Kober et al. additionally observed a more pronounced secretion of apoM to the brain parenchymal side (basolateral side) as to plasma, comparable to apo-AI [144,145] and PLTP [146]. We therefore hypothesize that S1P containing HDL-apoM particles are taken up by the BBB, followed by secretion to the brain parenchyma, intracerebral transport and S1P release. Rapid apoM exchange between HDL and VLDL/LDL particles has been reported [15,16] and peripheral derived apoM may be therefore transferred from apoA-I rich HDL particles to cerebral apoE rich HDL to enhance transport efficiency and S1P delivery.

Latest, Yanagida et al. highlighted the role of S1P_1_ in BBB permeability regulation [147]. Endothelial-specific S1P_1_ knockout mice display a significantly increased brain extravasation of administered tracers up to 3 kDa. Moreover, application of FTY720, a synthetic analogue of S1P and inducer of S1P_1_ internalization upon binding, partially recapitulated the phenotype in WT animals. Here, the authors detected cerebral extravasation of an applied 1 kDa tracer molecule 3 days post consecutive FTY720 treatment and further observed a decline of the effect after 7 days. Comparable experiments with 3 kDa sized traces are however absent. Elevation of apoM bioavailability and thereby S1P may be a potential therapeutic approach to significantly enhance the transport of CNS targeted drugs across the BBB to render treatment of various brain associated pathologies.

## 9. Conclusions

The discovery of apoM revealed a crucial element in transport of S1P and also suggests a unique role of apoM in distinct organ systems. Whereas hepatic apoM shuttles S1P through the blood circulation to different cellular networks, kidney derived apoM acts as a scavenger to prevent urinal S1P loss. The role of BBB derived apoM is essentially unknown to date. Several reports identified a link between the apoM/S1P system and liver fibrosis as well as brain inflammation. Reports on HDL-apoM and S1P receptor mediated effects on liver regeneration are controversial, but a unique role of apoM may be evident. ApoM further exerts a crucial role in minimizing CNS associated inflammatory processes in respective model systems. Whether the apolipoprotein is directly involved or serves a secondary role needs to be addressed in further studies. Moreover, modulation of the apoM/S1P system may be an efficient strategy to enhance CNS directed drug transportation via temporal BBB opening.

## Figures and Tables

**Table 1:** Average K_D_ values of S1P binding to various acceptor proteins. RApoM: recombinant human Apolipoprotein M; ApoD: human Apolipoprotein D; RBP: human retinol binding protein; SA: human Serum Albumin.

Ligand	ApoM	ApoD	RBP	SA
Retinoic acid	1.8 µM [18]	4.0 µM [19] ~2.7 µM [28]	0.21 µM [20] ~0.18 µM [28]	33.3 µM [29]
Retinol	2.2 µM [18]	0.2 µM [19] ~0.08 µM [28]	0.19 µM [20] ~0.26 µM [28]	13.2 µM [29]
S1P	rApoM ~0.9 µM [8] HDL associated 0.021 µM [24] LDL associated 0.0024 µM [24]	N/A	N/A	22.0 µM [24]

## Abbreviations

apoD	Apolipoprotein D
apoE	Apolipoprotein E
apoM	Apolipoprotein M
BBB	Blood-Brain-Barrier
BDL	Bile duct ligation
CCl_4_	Carbon tetrachloride
CNS	Central Nervous System
EAE	Autoimmune Encephalomyelitis
EC	Endothelial cell
ECM	Extracellular matrix
FOXA2	Hepatocyte nuclear factor 3-β
HDL	High Density Lipoproteins
HNF-1α	Hepatocyte Nuclear Factor-1α
HSC	Hepatic stellate cell
LDL	Low Density Lipoprotein
LRP2	LDL receptor related protein 2 (Megalin)
pBCEC	porcine Brain Capillary Endothelial Cell
PKC	Protein kinase C
PLTP	Phospholipid Transfer Protein
RBC	Red blood cell
S1P	Sphingosine-1-Phosphate
S1P_1_	S1P receptor 1
SA	Serum albumin
SMA	Smooth muscle actin
SPHK	Sphingosine Kinase
SPNS2	Sphingolipid Transporter 2
SR-BI	Scavenger receptor class B member 1
TGF-β	Growth Factor β
VLDL	Very Low Density Lipoprotein
